# Qualitätssteigerung der Abrechnungsprüfung durch Smartphone-basierte Fotodokumentation in der Unfall‑, Hand- und plastischen Chirurgie

**DOI:** 10.1007/s00113-020-00866-8

**Published:** 2020-09-15

**Authors:** Martin C. Jordan, Sebastian Jovic, Fabian Gilbert, Andreas Kunz, Maximilian Ertl, Ute Strobl, Rafael G. Jakubietz, Michael G. Jakubietz, Rainer H. Meffert, Konrad F. Fuchs

**Affiliations:** 1 Klinik und Poliklinik für Unfall‑, Hand‑, Plastische und Wiederherstellungschirurgie, Universitätsklinikum Würzburg, Oberdürrbacher Straße 6, 97080 Würzburg, Deutschland; 2 Institut für Diagnostische und Interventionelle Radiologie, Universitätsklinikum Würzburg, Oberdürrbacher Straße 6, 97080 Würzburg, Deutschland; 3 Servicezentrum Medizin-Informatik (SMI), Universitätsklinikum Würzburg, Schweinfurter Str. 4, 97080 Würzburg, Deutschland; 4 Verwaltung, Referat 3.3.2 – Medizincontrolling, Universitätsklinikum Würzburg, Josef-Schneider-Straße 2, 97080 Würzburg, Deutschland

**Keywords:** Digitalisierung, Gesundheits-App, Künstliche Intelligenz, Plattform, Strukturwandel, Artificial intelligence, Database, Digital transformation, Photo app, Surgery

## Abstract

**Hintergrund:**

Die Fotodokumentation von offenen Frakturen, Wunden, Dekubitalulzera, Tumoren oder Infektionen ist ein wichtiger Bestandteil der digitalen Patientenakte. Bisher ist unklar, welchen Stellenwert diese Fotodokumentation bei der Abrechnungsprüfung durch den Medizinischen Dienst der Krankenkassen (MDK) hat.

**Fragestellung:**

Kann eine Smartphone-basierte Fotodokumentation die Verteidigung von erlösrelevanten Diagnosen und Prozeduren sowie der Verweildauer verbessern?

**Material und Methoden:**

Ausstattung der Mitarbeiter mit digitalen Endgeräten (Smartphone/Tablet) in den Bereichen Notaufnahme, Schockraum, OP, Sprechstunden sowie auf den Stationen. Retrospektive Auswertung der Abrechnungsprüfung im Jahr 2019 und Identifikation aller Fallbesprechungen, in denen die Fotodokumentation eine Erlösveränderung bewirkt hat.

**Ergebnisse:**

Von insgesamt 372 Fallbesprechungen half die Fotodokumentation in 27 Fällen (7,2 %) zur Bestätigung eines Operationen- und Prozedurenschlüssels (OPS) (*n* = 5; 1,3 %), einer Hauptdiagnose (*n* = 10; 2,7 %), einer Nebendiagnose (*n* = 3; 0,8 %) oder der Krankenhausverweildauer (*n* = 9; 2,4 %). Pro oben genanntem Fall mit Fotodokumentation ergab sich eine durchschnittliche Erlössteigerung von 2119 €. Inklusive Aufwandpauschale für die Verhandlungen wurde somit ein Gesamtbetrag von 65.328 € verteidigt.

**Diskussion:**

Der Einsatz einer Smartphone-basierten Fotodokumentation kann die Qualität der Dokumentation verbessern und Erlöseinbußen bei der Abrechnungsprüfung verhindern. Die Implementierung digitaler Endgeräte mit entsprechender Software ist ein wichtiger Teil des digitalen Strukturwandels in Kliniken.

## Abstract (en)

**Background:**

Photographic documentation of wounds, decubitus ulcers, tumors, open fractures and infections is an important part of digital patient files. It is unclear whether the photographic documentation has an effect on medical accounting with health insurance companies.

**Objective:**

It was hypothesized that Smartphone-based systematic photographic documentation can improve the confirmation of proceeds-relevant diagnoses and procedures as well as the duration.

**Material and methods:**

Staff in the emergency room, operating theater, outpatient clinic and on the wards were equipped with digital devices (Smartphone, tablet) including a photo-app. Medical accounting with the health insurance companies and identification of all case conferences in which the photographic documentation had effected a change in proceeds were analyzed for 2019 in a retrospective manner.

**Results:**

Overall, 372 cases were discussed of which 27 cases were affected by the digital photographic documentation. Photographic documentation was used for clarification of the operative procedure (*n* = 5), primary diagnosis (*n* = 10), secondary diagnosis (*n* = 3), and length of hospitalization (*n* = 9). An average of 2119 € was negotiated and added per case affected by photographic documentation. Hereby, a level 1 trauma center gained an estimated 65,328 € in revenue.

**Discussion:**

The use of Smartphone based photographic documentation can improve the overall quality of patient files and thus avoid loss of revenue. The implementation of digital devices with corresponding software is an important component of the digital structural change in hospitals.

## Einleitung

Eine gut nachvollziehbare Dokumentation über Verletzungen, durchgeführte Operationen, vorliegende Haupt- und Nebendiagnosen sowie die Wundheilung während des stationären Aufenthalts ist für die Abrechnung zwischen Kliniken und Kostenträgern essenziell. Neben dem schriftlichen Befund und der radiologischen Bildgebung ist die digitale Fotodokumentation von Verletzungen und Wunden mittlerweile ein zentraler Bestandteil der Patientenakte. Derzeit ist unklar, welchen Stellenwert die digitale Fotodokumentation bei der Abrechnungsprüfung mit dem Kostenträger hat. Deshalb ist das Ziel dieser Studie, den Zusammenhang zwischen der Abrechnungsprüfung und einer Smartphone-basierten Fotodokumentation zu analysieren. Die Arbeitshypothese lautet, dass die systematische Fotodokumentation einen relevanten Einfluss auf die Dokumentation des klinischen Verlaufs hat und somit die korrekte Kodierung und Kostenabrechnung unterstützt. Dies kann dem Leistungserbringer bei Rechnungsprüfung mit dem Kostenträger helfen, den Erlös komplexer chirurgischer Fälle realistisch zu belegen.

## Material und Methoden

### Smartphone-basierte Fotodokumentation

**Figure 1:**
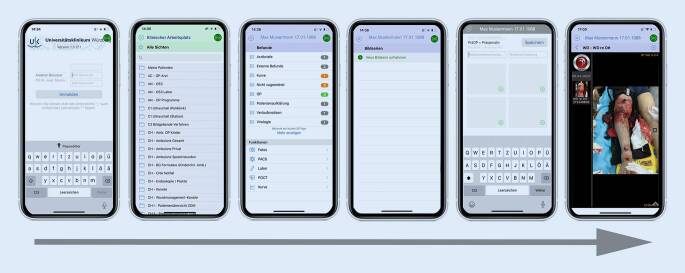

Im Rahmen eines von der Landesregierung finanzierten Digitalisierungsprojekts am Universitätsklinikum Würzburg erfolgte zunächst die eingeschränkte Ausgabe von Tabletcomputern (iPad Generation 3–4; Fa. Apple, Cupertino, Kalifornien, USA) an ärztliches und pflegerisches Personal, um mit einer eigens konzipierten App auf Patientendaten im Kliniknetzwerk zugreifen zu können (UKW-Mobile App, SMI, Würzburg; Abb. 1). Diese App erlaubt den Zugriff auf alle Patientendokumente wie z. B. Arztbriefe, Untersuchungsbefunde oder auf die radiologische Diagnostik. 2016 wurde diese Applikation um die Möglichkeit einer fallverknüpften Fotodokumentation erweitert. Hierbei werden die gewonnenen Fotos direkt mit Notizen im Picture Archiving and Communication System (PACS, Fa. Phönix PACS GmbH) gespeichert und sind somit ebenfalls jederzeit dezentral und sicher abrufbar. Durch das Digitalisierungszentrum für Präzisions- und Telemedizin (DZ.PTM) erfolgte ab 2018 u. a. die schrittweise Ausgabe von iPhones XR (Fa. Apple) an ärztliche Mitarbeiter zur Ausweitung der Fotodokumentation und besseren Implementierung der App in den klinischen Alltag. Im Jahr 2019 wurden alle Mitarbeiter der Klinik und Poliklinik für Unfall‑, Hand‑, Plastische und Wiederherstellungschirurgie aufgefordert, die App zur Fotodokumentation in der Notaufnahme, im OP und im Schockraum zu verwenden. Verletzungen, Ausgangsbefunde, Dekubiti, Wunden, Wundheilungsstörungen oder Infektionen wurden isoliert abgebildet und in das System eingespeist. Mit dieser Technologie ergibt sich die Möglichkeit, eine qualitativ hochwertige, systematische Fotodokumentation bei entsprechender Indikation vorzunehmen.

### Datenschutz

Die Berücksichtigung sämtlicher datenschutzrechtlicher Aspekte bei der Arbeit mit Patientendaten stellt im Alltag eine zwingende Voraussetzung dar. Die UKW-Mobile App ist eine Entwicklung der klinikeigenen IT-Abteilung und berücksichtigt die Datenschutz-Grundverordnung (DSGVO). Fotodokumente werden nach Auswahl des betroffenen Patienten innerhalb der App erstellt und nicht auf dem Gerätespeicher abgelegt. Somit wird verhindert, dass Dokumente unbeabsichtigt in nichtklinischen Cloud-Systemen hinterlegt werden. Ein Upload erfolgt ausschließlich auf das klinikeigene Serversystem. Wenn ein Patient auf einem Foto eindeutig zu erkennen ist, muss vor einer Verbreitung dieser Bilder eine Einwilligung eingeholt werden (Vorträge, Publikationen). In Fällen, in denen eine Zuordnung z. B. aufgrund einer sehr individuellen Verletzung möglich scheint, empfiehlt es sich ebenfalls, eine Einwilligung einzuholen. Es sollte immer berücksichtigt werden, dass die Fotodokumentation einer hilfsbedürftigen Person ohne medizinisches Ansinnen strafbar ist.

### Analyse der Abrechnungsprüfung

Mithilfe des Medizincontrollings, des DRG-Beauftragten und den DRG-Assistenten erfolgte eine retrospektive Auswertung der MDK-Begehungen in der Unfall‑, Hand- und plastischen Chirurgie der Monate Januar bis Dezember 2019. Grunddokument der Auswertung waren die vorliegenden Besprechungsprotokolle, aus denen die Argumentation und Begründung aller behandelter Fälle hervorgeht. Die anschließende Auswertung der Daten erfolgte pseudonymisiert. Ein Ethikvotum war dementsprechend nicht erforderlich. Von allen veröffentlichen Bildern liegt die unterschriebene Einwilligung betroffener Personen vor. Der klinische Verlauf der diskutierten Fälle wurde analysiert. Fälle, in denen eine Fotodokumentation eine OPS oder Diagnose sichern konnte, wurden herausgegriffen und die Veränderung der Abrechnung dokumentiert. Hierbei wurde zwischen einem erlösmindernden, einem erlössteigernden oder einem gleichbleibenden Effekt unterschieden. Der monetäre Gegenwert der erhaltenen oder verlorenen Diagnose bzw. Prozedur wurde erhoben und abschließend summiert (Internationale statistische Klassifikation der Krankheiten und verwandter Gesundheitsprobleme, 10. Revision, German Modification, Version 2019 mit Aktualisierung vom 01.11.2019 [ICD-GM-10 2019] sowie Operationen- und Prozedurenschlüssel, Version 2019 mit Aktualisierungen bis zum 03.12.2018 [OPS-Version 2019], beide DIMDI sowie ID Diacos; ID Information und Dokumentation im Gesundheitswesen GmbH & Co. KGaA).

## Ergebnisse

Im Jahr 2019 erfolgten 372 Fallbesprechungen zwischen dem Universitätsklinikum Würzburg und dem Medizinischen Dienst der Krankenkassen (MDK). Hierbei handelte es sich nur um Patienten aus der Unfall‑, Hand- und plastischen Chirurgie. Von 372 Fällen lag bei 134 (36 %) eine Fotodokumentation im System vor. In 27 (7,2 %) Fällen half die Fotodokumentation zur Sicherung/Rechtfertigung/Verteidigung entweder einer OPS (*n* = 5; 1,3 %), einer Hauptdiagnose (*n* = 10; 2,7 %) oder Nebendiagnose (*n* = 3; 0,8 %) sowie der Krankenhausverweildauer (*n* = 9; 2,4 %). Pro oben genanntem fotodokumentierten Fall ergab sich eine durchschnittliche Erlössteigerung von 2119 €. Die Summe aller Fälle erzeugte eine Erlössteigerung von 57.228 €. Je verhandeltem Fall wurde noch eine Aufwandspauschale von 300 € addiert, womit sich abschließend eine Gesamtsumme von 65.328 € für das Jahr 2019 ergab (Tab. 1).

**Table 1:** 

Fall	Diagnose	Fotodokumentation	MDK	Erlös
1	Quetschwunde, Hand Durchtrennung GNB 6	Risswunde, D III, beugeseitig	Verweildauer	671 €
2	Risswunde, Oberarm	Großflächige Wunde	Hauptdiagnose	2658 €
3	Implantatinfektion Paraplegie	Wunden am Fuß	Nebendiagnose (Dekubitus)	Keine Änderung
4	Schnittverletzung, Unterarm	Verzögerte Wundheilung	Verweildauer	671 €
5	Amputation, Daumen	Amputat (Nachweis einer vollständigen Amputation)	Hauptdiagnose	8217 €
6	Schnittverletzung, Hand mit Durchtrennung von A. und N. ulnaris und FCU	Schnittverletzung, Unterarm	Hauptdiagnose	1380 €
7	Pertrochantäre Femurfraktur	Dekubitus	Nebendiagnose (Dekubitus)	Keine Abzüge
8	Dekubitus	Wundheilungsstörung	Verweildauer	709 €
9	Schnittwunde am Fuß	Risswunde	Verweildauer	1260 €
10	Offene Unterschenkelfraktur	III° offene Fraktur	Hauptdiagnose	Keine Abzüge
11	Bursitis olecrani	Infizierte Bursa	Hauptdiagnose	Keine Abzüge
12	Fettschürze, Abdomen	Befunddokumentation	Hauptdiagnose	3366 €
13	Risswunde, Handrücken mit Strecksehnenverletzung	Risswunden am Handrücken	Verweildauer	Keine Abzüge
14	Abdominoplastik	Fettschürze	Verweildauer	2065 €
15	Unterschenkelamputation mit Wundheilungsstörung	Nekrose am Stumpf	Verweildauer	729 €
16	Amputation, D II–V	Amputat (Nachweis der Replantation)	OPS	9129 €
17	Infiziertes Atherom	Infektfokus	OPS	Keine Änderung
18	Dermatochalasis	Präoperative Weichteildokumentation	Verweildauer	424 €
19	Dekubitus	Offene Wunde, glutäal (Débridement >4 cm^2^)	OPS	2826 €
20	Periprothetische Femurfraktur	Dekubitus	Nebendiagnose	Keine Abzüge
21	Pseudarthrose, Unterschenkel	Spalthautdeckung	OPS	Keine Abzüge
22	Wundheilungsstörung, Unterschenkel	Defektausmaß	Hauptdiagnose	1760 €
23	Adipositas, geplanter „body lift“	Fettschürze	Hauptdiagnose	806 €
24	Mittelhandamputation	Stumpf (Nachweis der Amputation)	Hauptdiagnose	7441 €
25	Verschleppter Infekt am Fuß nach Stichverletzung	Aufnahmebefund mit Stichverletzung und septischem Fuß	Hauptdiagnose	7201 €
26	Schnittverletzung, Hohlhand	Unfallbilder	Verweildauer	3025 €
27	Osteomyelitis, Unterschenkel, behandelt mit freiem Lappen	Defektgröße	OPS	2890 €
Zwischensumme				57.228 €
Aufwandpauschale je Fall (300 €)				8100 €
*Gesamtbetrag*				*65.328* *€*

*GNB* Gefäß-Nerven-Bündel, *FCU* M. flexor carpi ulnaris

## Diskussion

**Figure 2:**
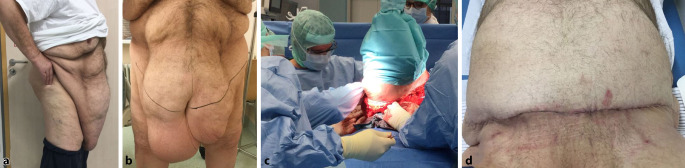

Insgesamt ist die Datenlage zu Abrechnungsprüfungen durch den MDK in Kliniken spärlich. Unstrittig ist aber, dass durch die Abrechnungsprüfung relevante Erlöskürzungen durch lückenhafte Dokumentation möglich sind. So hat 2018 der MDK Nordrhein 17 % aller Krankenhausfälle geprüft und in 50,3 % der Fälle eine Kürzung von durchschnittlich 2000 € pro Fall vorgenommen. Nur in 1 % der Fälle ergab sich eine Korrektur zugunsten der Krankenhäuser. Gegenstände der Prüfung waren in 57,8 % die stationäre Verweildauer und in 30,6 % die Kodierung [1]. Dieser Sachverhalt bestätigt die hohe Wertigkeit einer präzisen Dokumentation, wenn möglich durch die Verwendung klinischer Fotos. Im Vorfeld der Rechnungsprüfung werden üblicherweise fallspezifische Fragen der Krankenkassen durch den MDK an das Krankenhaus übermittelt. Neben den üblichen Dokumenten wie Ambulanz- und Entlassbrief, OP-Bericht, Labor- und Röntgenbefund können so im Vorfeld auch hilfreiche Fotodokumente aus dem Archiv bereitgestellt werden. Die hier vorgestellten Ergebnisse zeigen, dass eine Fotodokumentation insbesondere zur Bestätigung der Hauptdiagnose und zur Rechtfertigung der Verweildauer vorteilhaft ist. Es zeigt sich aber auch, dass die Fotodokumentation nur in relativ wenigen Fällen (7,2 %) einen Beitrag leisten kann. Auch wenn der Einsatz der Fotodokumentation ausgeweitet werden sollte, so bleibt diese Technik nur ein Baustein in der komplexen Abrechnungsprüfung. Eine gute schriftliche Dokumentation ist weiterhin unverzichtbar. Auffallend war in unserer Analyse, dass die Fotodokumentation überproportional häufig in der Hand- und Plastischen Chirurgie zur Klärung beitragen kann. Gerade in diesem Fachbereich hat die präoperative Fotodokumentation bei körperformenden Eingriffen eine lange Historie. Außerdem sollte der klinische Verlauf gut dokumentiert werden, um die Notwendigkeit weiterer Eingriffe oder der stationären Behandlung zu belegen. Insbesondere Weichgewebsveränderungen wie Wunden, Schwellungen und Infektionen sowie deren Verläufe können nicht durch Röntgendiagnostik oder Laborwerte nachvollzogen werden, weshalb die Fotodokumentation eine sinnvolle Ergänzung darstellt (Abb. 2 und 3). Unabhängig davon ist die Fotodokumentation möglicherweise bei juristischer Aufarbeitung nützlich [2].

**Figure 3:**
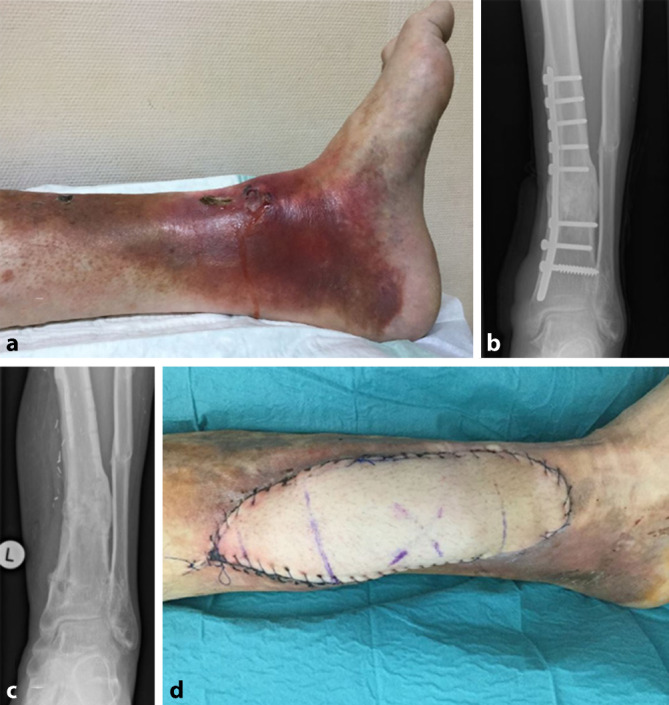

Vorteile der Smartphone-basierten Fotodokumentation mittels App sind, im Gegensatz zum Einsatz einer Digitalkamera, die einfachere Handhabung sowie die direkte Zuordnung zur Patientenakte. In der Vergangenheit war die Fotodokumentation aufgrund fehlender Geräte häufig nicht möglich [3]. Zudem gab es bei der Archivierung und Qualität der Fotos immer wieder Probleme. Wichtig ist eine konstante Qualität der Fotodokumentation, welche in Zukunft durch eine standardisierte Verfahrensanweisung weiter gesteigert werden kann [4, 5]. Hierfür ist die Entwicklung einer „standard operating procedure“ (SOP) mit definierten technischen Kriterien denkbar, wie z. B. gleichbleibendem Abstand und Winkel zum Objekt sowie einheitlicher Beleuchtung und abgebildeter Größenreferenz. Eine solche SOP wäre die Grundlage für die Anwendung einer automatisierten Auswertung (künstliche Intelligenz) und könnte die Abrechnung durch automatisierte Auswertung der Fotodokumentation weiter verbessern [6].

Ein weiterer Vorteil der systematischen Fotodokumentation ist die Verwendung der Bilder in der Ausbildung und Lehre. Außerdem kann die Bearbeitung von hausinternen oder sogar überregionalen Konsilanforderungen vereinfacht werden [7], insbesondere dann, wenn die Fotodokumentation delegierbar und eine persönliche Konsultation nicht erforderlich ist. Die Mitbeurteilung durch erfahrenere Kollegen oder andere Fachdisziplinen kann unter deutlicher Zeitersparnis erfolgen. Im Vergleich zur umständlichen und zeitraubenden Fotodokumentation mittels Digitalkamera ist die Benutzerfreundlichkeit („ease of use“) ein Vorteil, welcher zu mehr Akzeptanz und Einsatz führt. Keinesfalls darf der Austausch von Patientenbildern über ungeschützte Messenger-Dienste oder E‑Mail-Verkehr erfolgen.

Die Fotodokumentation offener Frakturen wird mittlerweile von nationalen und internationalen Fachgesellschaften dringend empfohlen [8]. Somit sollen eine unnötige mehrfache Abnahme des Verbands verhindert sowie die bessere Planung operativer Prozeduren ermöglicht werden. Des Weiteren kann die Fotodokumentation im Rahmen von Gutachten oder der Feststellung von Dauerschäden eine hilfreiche Ergänzung sein [9].

Ein Nachteil der am Universitätsklinikum Würzburg durchgeführten Art der Fotodokumentation sind die Anschaffungskosten der mobilen Endgeräte, der Software und der Unterhaltungskosten. Die Vorteile der Smartphone-Nutzung wie z. B. Steigerung der Effizienz und Arbeitserleichterung gehen aber über die Fotodokumentation hinaus und sind Teil eines notwendigen digitalen Strukturwandels. So werden die Smartphones auch zur innerklinischen Kommunikation verwendet. Die Kosten für eine solche Infrastruktur sollten deshalb bei anstehenden Investitionsverhandlungen berücksichtigt werden, da diese in Zukunft auch von qualifizierten Arbeitnehmern eingefordert werden [10, 11].

Limitationen unserer Studie ergeben sich aus der retrospektiven Datenanalyse, welche das Risiko einer Fehlinterpretation birgt. Ein prospektives Studiendesign mit Protokoll, definierten Einschlusskriterien, einer Vergleichsgruppe mit Randomisierung und festgelegten Outcome-Parametern kann die Aussagekraft zukünftiger Studien deutlich verbessern. Ein Kritikpunkt bezüglich der Smartphone-Nutzung im Krankenhaus ist abschließend erwähnenswert. Erkenntnisse aus der privaten Smartphone Anwendung deuten auf psychosoziale Risiken dieser Technologie hin, und der Einsatz am Arbeitsplatz ist umstritten [12]. Permanente Ablenkung durch die Smartphone-Nutzung während wichtiger Besprechungen, Beschäftigung mit dem Smartphone anstatt mit dem Patienten während der Visite oder kommentarlose Fotodokumentation ohne den expliziten Hinweis darauf, dass es sich nicht um ein privates Gerät handelt, können nach eigener Beobachtung zu Konflikten mit Patienten oder Kollegen führen.

## Fazit für die Praxis

- Die Smartphone-basierte Abbildung von Wunden oder intraoperativen Befunden kann die Qualität der Dokumentation verbessern.
- Bei der Abrechnungsprüfung hilft die systematische Fotodokumentation, Erlöseinbußen zu verhindern.
- Die Fotodokumentation erleichtert und beschleunigt die Abrechnungsprüfung.
- Die Implementierung digitaler Endgeräte mit entsprechender Software ist ein wichtiger Bestandteil des digitalen Strukturwandels in Kliniken.
